# The effect of additional shading utilizing agriphotovoltaic structures on the visual qualities and metabolites of broccoli

**DOI:** 10.3389/fpls.2023.1111069

**Published:** 2023-03-03

**Authors:** Hyeon-Woo Moon, Kang-Mo Ku

**Affiliations:** ^1^ Department of Horticulture, Chonnam National University, Gwangju, Republic of Korea; ^2^ Department of Plant Biotechnology, Korea University, Seoul, Republic of Korea

**Keywords:** broccoli, agriphotovoltaic, shading, appearance quality, metabolites

## Abstract

Agriphotovoltaic (APV) systems allow the simultaneous production of crops and electricity in the same land area. Since the reduction of yield caused by APV systems is important for food security, studies to improve the yield have been conducted steadily. However, there have been limited data on the appearance, quality, and metabolomic changes of crops. Therefore, in this study, we evaluated the visual qualities and metabolites as well as the yield of broccoli grown using an APV system during the fall season. In addition, additional shading treatment was performed, and the same qualities were evaluated. In the spring season, an additional cultivar that does not express anthocyanins was cultivated. Glucosinolate content was more sensitive to the seasonal environment and the type of cultivar than it was to treatment type. The additional shading treatment had a positive effect on the visual qualities of anthocyanin-expressing broccoli cultivar regardless of the season, and we observed that even a cultivar that does not express anthocyanins can be greener. Regardless of cultivar, higher chlorophyll content was detected in broccoli florets with additional shading treatment under the APV system. In addition, reduced anthocyanin content was observed (6.1 mg g^-1^ DW; about 20% of that obtained on open-field). Aspartic acid content was enhanced upon additional shading treatment. Pathway analysis revealed changes in anthocyanin, alanine, aspartic acid, and glutamic acid metabolism. Overall, our findings suggests that it is possible to produce crops with better visual qualities by utilizing APV systems.

## 1 Introduction

The agrivoltaic (AV) or agriphotovoltaic (APV) system, a term formed by the combination of agriculture and photovoltaic (PV), is a structure that allows the production of electricity above (on its surface) and the growth of crops below (located under it). This system coproducing food and energy was devised by Goetzberger and Zastrow and specifically designed by Dupraz et al. (Goetzberger and Zastrow, 1982; Dupraz et al., 2011; Luo et al., 2019). PV panels are staggered to reduce the amount of light reaching the plants. Shading is generally known to delay plant growth and development except in the leaf area (Díaz-Pérez, 2013). Growth and development are crucial as they directly influence the yield of horticulture crops. Therefore, research on crop yield of plants grown using APV systems has been conducted. Touil et al. (2021) reviewed the effect to yield of crop according to the type of crops, the cover ratio of panels, and region under the agriphotovoltaic system. Kumpanalaisatit et al. (2022) also reported yields of some species of plants grown with APV systems.

Despite the risk of decreased yield, APV systems are advantageous for the following reasons. Most importantly, APV systems can increase farmers’ profits, because they produce electricity (Chae et al., 2022). Plants with low light saturation points can thrive under low light conditions, because they cannot utilize light beyond the saturation point for photosynthesis (Tazawa, 1999). Excessive levels of light containing ultraviolet wavelengths could harm plants by damaging the photosynthetic system (Zlatev et al., 2012; Wimalasekera, 2019). Shading usually does not have a positive effect on overall production or quality; however, sometimes it could be used strategically to improve the quality of plant products. In the case of green tea, high-quality leaves called “Tencha” are produced by applying shading on green tea. For these reasons, as long as there is no significant impact on growth and yield, crops could be grown using APV systems (Ku et al., 2010).

Shading by APV structures could enhance crop quality to meet consumers’ visual preferences. Broccoli (*Brassica. oleracea* var. *italica*) contains health-promoting compounds such as glucosinolates, carotenoids, and polyphenols (Liu et al., 2018a). Among the secondary metabolites of broccoli, sulforaphane, a hydrolysis product of glucoraphanin, is known to exhibit anticancer activity (Ku et al., 2014). Although anthocyanins are health-promoting compounds, the color of broccoli head turns slightly red or purple when exposed to light. A simple survey we performed (data not shown) showed that when presented with green broccoli grown under an APV system and slightly purple-colored broccoli grown on open-field (OF), 93% of the participants preferred the former. In the case of cauliflower, studies have shown that both the yield and quality are increased through shading (Ismail and Ann, 2001; Sawant and Bichkule, 2018). Therefore, in commercial production, cauliflower is wrapped by their leaves a few weeks before harvest to create shade to improve quality. However, creating shading is not a feasible option for broccoli production under normal open-field conditions.

To our best knowledge, most APV system-related research has been focused on the crop yields to date. In addition, no studies have been conducted on the related applications of columns that are installed as a part of the photovoltaic systems. The effect of shading on broccoli physiology and associated metabolomic changes have not been intensively investigated. We previously conducted experiments to test the effects of APV systems on some metabolites and sensorial quality of cabbage (Moon and Ku, 2022). Therefore, in this study, we tested the effects of APV systems on other *B. oleracea* crops. In the spring of 2021, through a preliminary experiment, the difference in color of broccoli heads grown from open-field and agriphotovoltaic with a stepped degree of shading treatment was confirmed. After that, this experiment was conducted in the autumn with one specific cultivar, and the following spring, a non-anthocyanin-expressed cultivar was added to the same experiment to confirm whether it had a difference in color and metabolites by shading treatment or not. Here, we report pigment compounds and overall metabolomic changes induced by APV systems and by additional shading, utilizing an APV structure in two cultivars of broccoli for two seasons.

## 2 Materials and methods

### 2.1 Cultivation and environmental data collection

In 2021, ‘Earlyyou’ (Asia Seed Co., Ltd., Seoul, South Korea) broccoli was sown on July 26 in 105-cell seedling trays. On August 31, the seedlings were transplanted at the site with coordinates 34°58′28.4″N, 126°45′59.3″E (Naju, Jeollanam Province, Republic of Korea). ‘Earlyyou’ and ‘Youil2ho’ (Sakata Korea Co., Ltd., Seoul, Republic of Korea) broccoli were sown on May 2nd, transplanted on 12/04/2022, in an area equal to that in the previous cultivation. Planting distance and the space between the rows were 50 cm. A NovaTec Suprem (N:P:K; 21:5:10) controlled release fertilizer (Compo Expert, Münster, Westphalia, Germany) was sprinkled on the bed for supplying nutrients. Broccoli was harvested from 29/10/2021 to 23/11/2021, and two broccoli cultivars were harvested from 08/06/2022 to 13/06/2022.

The microclimate data during the cultivation period were collected by data loggers (ZL6, METER Group Inc., Pullman, WA, USA) placed on the OF and APV shading areas. The loggers had sensors to monitor air conditions (ATMOS14), soil environment (TEROS11), and photosynthesis active radiation (PAR). Soil sensors were placed 20 cm below the surface. All environmental data were collected at 10-min intervals.

### 2.2 Agriphotovoltaic structure

The 3.3-m-high columns of the APV structure were arranged at intervals of 4 × 5 m relative to the south. Two types of bi-facial PV panels (JAM60D09 320/BP, JA Solar Holdings, Beijing, China; LR6-60BP-310M, Longi Solar, Xi’an, China) were used. The length and width of the panels were 1.67 and 1 m, respectively. The spacing between the panels was 0.32 and 1.38 m, with respect to the south. The angle between the horizontal column from the ground and PV panel was 35°.

### 2.3 Shading treatment using the agriphotovoltaic structure

**Figure 1:**
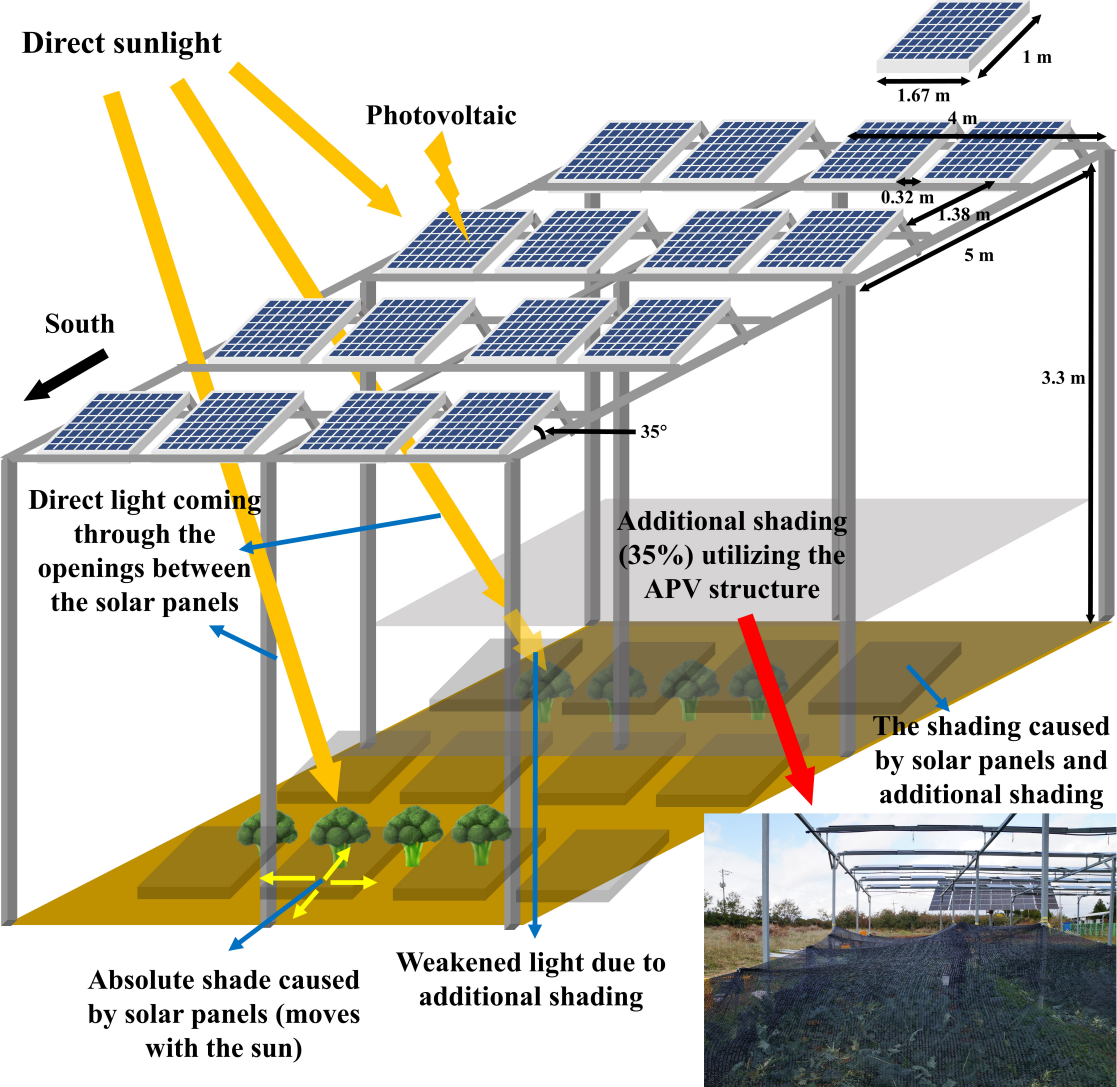
Schematic diagram of the agriphotovoltaic structure and the setting for additional shading treatment. Direct sunlight reaching to the panels is converted to electricity through the process of “photovoltaics.” The light entering through the gaps between the panels reach directly to the plants. When sunlight is blocked by the panels or other parts of the APV structure, an absolute shade is formed. The absolute shade moves as the relative position of the sun changes during the day. The shading curtain made of high-density polyethylene film is installed on the structure and covers the plants entirely during the head extension period of broccoli. The curtain provides additional shading and blocks about 35% of the direct sunlight.

For additional shading, 35% shading curtain made of high-density polyethylene (HDPE) film was used on the crops from 17/10/2021 to 23/11/2021 (**Figure 1**). The same type of film was used for shading from 08/06/2022 to 13/06/2022. The curtain was installed 150 cm above the ground and fastened to the posts with ropes. The shading was applied from the time when the head size of 2 cm exceeded 50% of the total until the end of the harvest.

### 2.4 Measurement of weight and diameter and the processing for metabolite analysis

Harvested broccoli was cut into 8-cm-long pieces. Then, the largest diameter of the stem was measured excluding the petiole. Broccoli heads were cut into small pieces and used to prepare freeze-dried samples. Fresh broccoli heads were dipped into liquid nitrogen (–196°C). Frozen broccolis were stored in a deep freezer (–80°C) and freeze-dried (–80°C, 5 mT) in a MCFD8508 freeze dryer (ilShinBioBase Co., Ltd., Dongducheon, Korea). The broccoli buds came apart from broccolis harvested in spring 2022 after freeze-drying to evaluate anthocyanin and chlorophyll contents.

### 2.5 Analysis of water-soluble primary metabolites

For detecting changes in several metabolite content approximately, water-soluble primary metabolites (some amino acids, organic acids, and sugar) were analyzed by gas chromatography (Nexis GC-2030, Shimadzu, Kyoto, Japan) with a gas chromatograph-mass spectrometer (GC/MS-QP 2020 NX, Shimadzu, Kyoto, Japan). The overall process was conducted as described previously (Moon and Ku, 2022). For the first step, freeze-dried powder (50 mg) was extracted in methanol (1.5 ml). Derivatization of water-soluble compounds was conducted by trimethylsilyl-N-methyl trifluoroacetamide (MSTFA). Methoxamine was added before derivatization to prevent the formation of multiple derivatives during silylation. Metabolites were identified based on the database of the National Institute of Standards and Technology (NIST).

### 2.6 Determination of amino acids

The amino acids were analyzed according to the manufacturer’s protocol (AdvanceBio Amino Acid Analysis, Agilent Technologies, PA, USA). Freeze-dried powder (50 mg) was extracted in 1 ml of 0.1 N HCl solution at 800 rpm for 10 min. The solvent was centrifuged at 12,000 × *g* for 3 min. The supernatant was filtered through a 0.22-µm nylon syringe filter and poured into a vial. The chromatographic separation was performed by Agilent 1100 HPLC equipped with a UV-vis detector and C18 reversed-phase column (HSS T3, Waters, Milford, MA, USA; 100 mm × 3 mm, 3.5 µm, 100 Å). Identification and quantification of amino acids were conducted using authentic amino acid standards.

### 2.7 Analysis of glucosinolates and hydrolysis products

Freeze-dried broccoli powder was extracted with 2 ml of 70% methanol at 95°C for 10 min. After centrifugation at 2,000 × *g* for 10 min, 500 μl of 1 mM glucosinalbin (isolated from the seeds of *Sinapis alba*) was added as the internal standard. The supernatant was poured into a new tube, and one more extraction was conducted without the addition of the internal standard. The extracted solution (1 ml) was mixed with 150 μl of 0.5 M lead barium acetate solution. After centrifugation at 12,000 × *g* for 2 min, the mixture was passed through a poly-prep column charged with resin (DEAE Sephadex A-25, GE Healthcare, Piscataway, NJ, USA). Then, 3 ml of 0.02 M pyridine acetate and 3 ml of distilled water were drained through the column. The column was sealed by a cap immediately after pouring 500 μl of sulfatase solution (20 U ml^-1^, *Helix pomatia* Type H-1, Sigma-Aldrich, St. Louis, MO, USA) and incubated for 16 h at room temperature. The solution containing desulfo-glucosinolates was injected into high-performance liquid chromatography (Agilent 1100, Agilent Technologies, PA, USA), fitted Kromasil^®^ reversed-phase C18 column (250 mm × 4.6 mm, 5 µm, 100 Å). The mobile phase A and chromatography separation was performed with gradient as follows: 0 min, 0.5% B; 4.5 min, 3% B; 7 min, 15% B; 24 min 25% B; 25 min, 100% B; 27 min, 100% B; 28 min, 0.5% B; 30 min, 0.5% B. For identified glucosinolates, an ultra-high-performance liquid chromatograph (ACQUITY Arc, Waters, Milford, MA, USA) coupled with a single quadrupole mass detector was used.

Analysis of glucosinolate hydrolysis products was conducted using an identical method previously described without an incubation process(Moon and Ku, 2022). To put it briefly, freeze-dried broccoli powder was extracted in deionized water. Phenyl isothiocyanate was added as internal standard. The solution came together with dichloromethane and incubated for 16 h at room temperature. After centrifugation, the dichloromethane layer was used for analyzing by GC/MS.

### 2.8 Measurement of total anthocyanins and chlorophyll content

The total anthocyanins were detected by a pH differential method (Zhu et al., 2017). Broccoli buds (500 mg) were transferred into a 15-ml tube containing 10 ml of 50% methanol with 1% formic acid for extracting. The mixture was vortexed and vigorously shaken for 24 h at 4°C. The blended solution was centrifuged at 2,000 × *g*, and 200 μl of supernatant was mixed with 100 μl of pH 1.0 buffer (potassium chloride, 0.025 M) and pH 4.5 buffer (sodium acetate, 0.4 M). The absorbance of the mixture was measured using a UV spectrophotometer (SpectraMax ABS Plus, Molecular Devices, CA, USA) at 520 and 700 nm.

The total chlorophyll content was determined by colorimetric assay using the extracted solution of broccoli buds. Broccoli powder (75 mg) was combined with 1.5 ml acetone. The mixture was shaken for 24 h in the dark. Two more extraction steps were performed until the color of powder completely turned white. After centrifugation at 12,000 × *g* for 3 min, the absorbance of the supernatant was measured by a UV spectrophotometer at 645 and 663 nm.

### 2.9 Color measurement

The colors of the broccoli heads were determined by a colorimeter (NR60CP, Shenzhen 3nh Technology Co., Ltd., Shenzhen, China). The colorimeter was calibrated by white (L*, 97.13; a*, 0.05; b*, –0.76) and black (L*, 0; a*, 0; b*, 0) standards. Four colorimetric parameters (L*, lightness; a*, redness; b*, yellowness; hue angle) were evaluated. Color parameters were measured from five random points per head, and 10 heads were regarded as one replicate.

### 2.10 Statistical and multivariate analyses

The statistical analyses were performed using the statistical software JMP 12. One-way analysis of variance (ANOVA) was performed to analyze differences among the three treatment groups. Tukey’s honestly significant difference (HSD) test was used for *post-hoc* analysis. Differences with *p <* 0.05 were considered significant. Multivariate analysis was performed using MetaboAnalyst 5.0 (https://www.metaboanalyst.ca).

## 3 Results

### 3.1 The microclimate of cultivation

The microclimate data collected after shading treatment are shown in **Table 1**. Regardless of the season, the light environment changed the most due to the APV system and shading treatment compared with the OF. In the fall of 2021, photosynthetic photon flux density (PPFD) in the APV system and shading treatment decreased by 42% and 59%, respectively, compared with the OF. In the spring of 2022, the PPFD in the APV system and shading treatment decreased by 30% and 62%, respectively. The average soil temperature decreased by 0.9°C upon shading treatment in the fall of 2021. On the other hand, the average soil temperature decreased by 0.8 and 1.0°C in the APV system and with shading in the spring of 2022, respectively.

**Table 1:** The mean microclimate data comparing the three growth conditions.

Year	Season	Treatment	PPFD (µmol m^-2^s^-1^)	Air temperature (°C)	Water content (%)	Soil temperature (°C)
2021	Fall	Open-field	532.6	9.9	30.1	12.6
		Agriphotovoltaic	309.7	9.8	30.7	12.6
		Shading	219.6	-	30.0	11.7
2022	Spring	Open-field	580.9	20.5	26.6	21.1
		Agriphotovoltaic	372.7	20.6	34.5	20.3
		Shading	200.4	20.4	-	20.1

Due to unknown reason of disconnection in the sensor, the air temperature of the 2021 fall shading and the water content of the 2022 spring shading were not properly recorded. The missing data were marked with the "-" symbol.

Due to an unknown reason of disconnection in the sensor, the air temperature of the 2021 fall shading and the water content of the 2022 spring shading were not properly recorded. The missing data were marked with the “-” symbol.

### 3.2 Changes in the average broccoli head weight under the APV system and upon additional shading treatment

**Figure 2:**
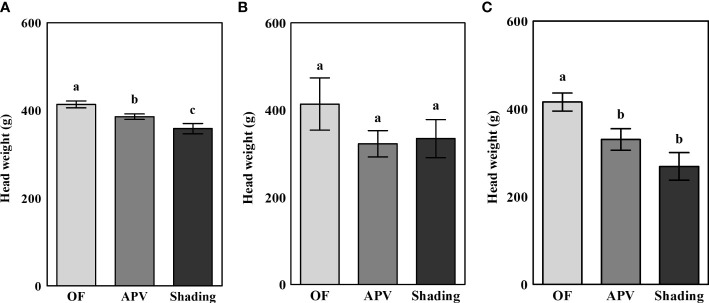
The average head weight of fall 2021 ‘Earlyyou’ **(A)**, spring 2022 ‘Earlyyou’ **(B)**, and ‘Youil2ho’ **(C)**. OF, open-field; APV, agriphotovoltaic system. Head weight was measured after cutting the stem length to 8 cm. The weight of 20 broccolis for each treatment was considered as one replication. Lowercase letters indicate significant differences among treatments within the same cultivar and year by HSD Tukey’s test (*n* = 3, *p* < 0.05).

The average head weights of broccoli cultivated under different conditions are shown in **Figure 2**. The head weight of the ‘Earlyyou’ cultivar showed significant differences among treatments in the fall of 2021, whereas no significant differences were detected in the spring of 2022. The ‘Youil2ho’ cultivar exhibited significant differences when grown under an APV system or additional shading; however, there were no differences between the plants grown under the APV system and those receiving additional shading treatment. Compared with those grown on OF, the head weights of plants grown under the APV system and with shading in 2021 fall decreased by 6.8% and 13.3%, respectively. In the spring of 2022, the corresponding decreases in head weights were as follows: ‘Earlyyou’, 22.0%, 19.3%; ‘Youil2ho’, 20.7% and 35.3%.

### 3.3 Changes in broccoli head color under the APV system and additional shading treatment

**Figure 3:**
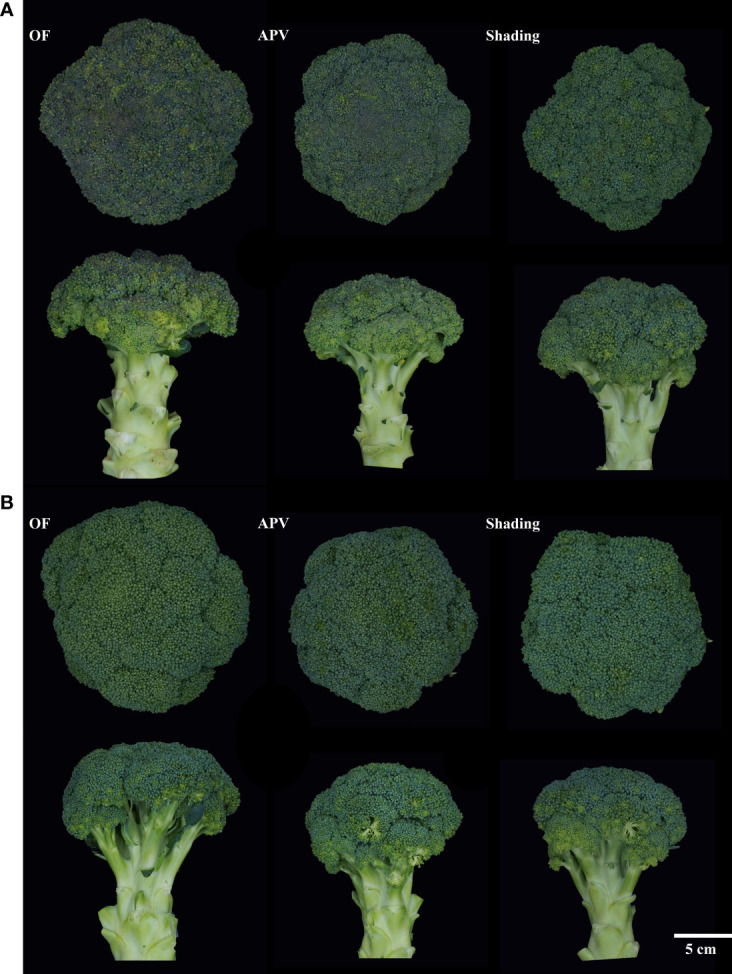
Representative photos for each treatment group (OF, open-field; APV, agriphotovoltaic) of spring 2022 ‘Earlyyou’ **(A)** and ‘Youil2ho’ **(B)**. Representative broccoli corresponding each treatment was expressed as picture of its shape of top (above) and side (bottom). There were no pictures in the fall of 2021, but similar tendency was emerged to spring of 2022 borccoli by treatments.

The representative images of broccoli heads are shown in **Figure 3**, and the assessment of color changes in broccoli heads is compiled in **Table 2**. L* of ‘Earlyyou’ significantly increased upon shading treatment, whereas ‘Youil2ho’ did not show significant changes. The colors of plants grown on OF or under the APV system significantly differed in the fall of 2021 but not in the spring of 2022. Independent of the season, a* of ‘Earlyyou’ was significantly lower in plants grown using the APV system and with shading compared with those grown on OF. On the other hand, the ‘Youil2ho’ cultivar did not show significant differences in a* values. A significant change in b* upon treatments of broccoli was only observed in ‘Earlyyou’ grown in the fall of 2021; b* levels were significantly higher in plants grown using the APV system compared with those grown on OF, and b* levels of those grown with shading were higher compared with those grown using the APV system. Hue angle showed significant changes among treatments regardless of season and cultivar by one-way ANOVA. In the ‘Earlyyou’ cultivar, the hue angle was significantly higher in plants grown using the APV system and with shading treatment compared with those cultivated on OF. Shading treatment in ‘Youil2ho’ resulted in a significantly higher hue angle compared with OF, whereas the APV system did not cause any difference compared with OF or shading.

**Table 2:** The color properties of broccoli according to season, cultivar, and treatment.

				CIELAB values			
Year	Season	Cultivar	Treatment	L*	a*	b*	Hue angle (˚)
2021	Fall	Earlyyou	Open-field	33.7 ± 3.3 b	-1.1 ± 0.2 a	9.1 ± 0.5 c	95.5 ± 1.9 b
			Agriphotovoltaic	36.6 ± 2.6 a	-5.1 ± 0.6 b	15.1 ± 0.5 b	108.9 ± 1.5 a
			Shading	39.1 ± 2.8 a	-6.3 ± 1.6 c	18.3 ± 1.0 a	109.2 ± 3.4 a
2022	Spring	Earlyyou	Open-field	36.4 ± 2.7 b	-4.3 ± 0.1 a	19.6 ± 1.1 a	103.2 ± 0.5 b
			Agriphotovoltaic	41.4 ± 1.5 ab	-6.4 ± 0.4 b	21.9 ± 1.9 a	107.6 ± 1.9 a
			Shading	44.1 ± 1.6 a	-8.6 ± 0.3 c	25.1 ± 4.3 a	110.2 ± 2.4 a
		Youil2ho	Open-field	41.2 ± 3.0 a	-8.5 ± 1.1 a	23.0 ± 5.2 a	111.8 ± 1.6 b
			Agriphotovoltaic	40.9 ± 1.1 a	-9.3 ± 0.2 a	23.0 ± 2.9 a	114.2 ± 2.3 ab
			Shading	43.3 ± 0.2 a	-9.6 ± 0.3 a	19.9 ± 2.7 a	117.4 ± 2.2 a

The data were expressed as the mean ± SD (n = 3). L*: lightness, a*: redness, b*: yellowness. Each color property of eight broccolis by treatment was considered as one replication. Lowercase letters indicate significant differences among treatments within the same cultivar and year by HSD Tukey's test (p < 0.05). The CIELAB values of fall of 2021 broccoli were published on our previous study (Chae et al., 2022).

The data were expressed as the mean ± SD (*n* = 3). L*: lightness, a*: redness, b*: yellowness. Each color property of eight broccolis by treatment was considered as one replication. Lowercase letters indicate significant differences among treatments within the same cultivar and year by HSD Tukey’s test (*p* < 0.05). The CIELAB values of fall of 2021 broccoli were published on our previous study (Chae et al., 2022).

### 3.4 The differences in metabolites due to the agriphotovoltaic system and shading compared with open-field

#### 3.4.1 Changes in metabolites

**Figure 4:**
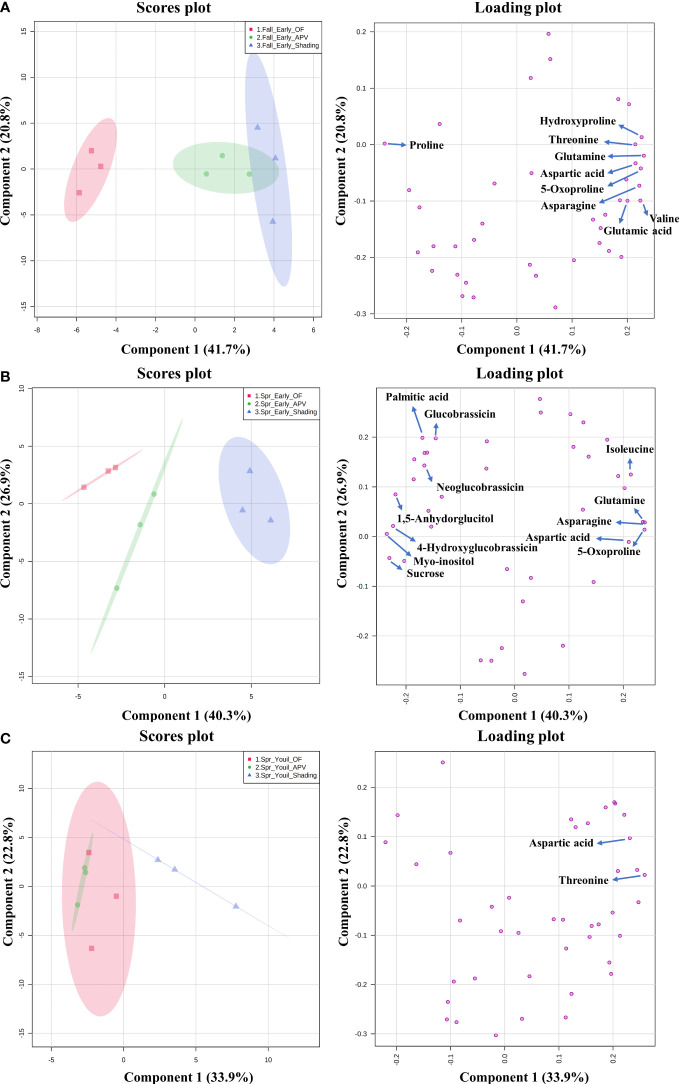
The principal component analysis (PCA) o analyzed metabolites from fall ‘Earlyyou’ **(A)**, spring ‘Earlyyou’ **(B)** and spring ‘Youil2ho’ **(C)** by treatment type. The squares, circles, and triangle on each graph indicate the replicate of OF, APV, and additional shading treatment in APV groups, respectively. The results are presented in score plots (left column) and loading plots (right column) to visually help distinguish among the groups and mark certain metabolites among the groups by HSD Tukey’s test marked as their compound name of the leading plot (p<0.05).

The analysis of water-soluble compounds tentatively identified 27 compounds with the NIST Library or standard compounds. The result of the principal component analysis (PCA) containing three groups and variables (water-soluble metabolites, glucosinolates, and amino acids) is shown in **Figure 4**. Among the compounds, the names of those that exhibited significant changes (as determined by one-way ANOVA) are marked on the loading plot. The score plot of ‘Earlyyou’ grown in the fall of 2021 showed that OF and shading were distinguished in the 95% confidence interval range, whereas the APV system was not separated from shading treatment by metabolites. The levels of hydroxyproline, proline, aspartic acid, glutamine, threonine, valine, asparagine, glutamic acid, and 5-oxoproline did not significantly differ among the groups. Among them, only proline levels were high in plants grown on OF, and the levels of other compounds were the highest in plants grown with shading treatment. In the spring of 2022, groups of each treatment were totally separated by relative amounts of compounds in the ‘Earlyyou’ cultivar. Asparagine, 5-oxoproline, 1,5-anhydroglucitol, neoglucobrassicin, myo-inositol, isoleucine, glutamine, sucrose, glucobrassicin, aspartic acid, hydroxyglucobrassicin, and palmitic acid levels were significantly different among groups. Among them, glucobrassicin, neoglucobrassicin, palmitic acid, 1,5-anhydroglucitol, 4-hydroxyglucobrassicin, myo-inositol, and sucrose levels were relatively high in plants receiving shading treatment, whereas isoleucine, glutamine, asparagine, aspartic acid, and 5-oxoproline levels were the highest in plants grown with shading treatment. Among the ‘Youil2ho’ cultivar groups, APV and shading treatment groups were separated. There were only two compounds (aspartic acid and threonine) with significantly different levels among the groups, which were high in plants receiving shading treatment.

**Figure 5:**
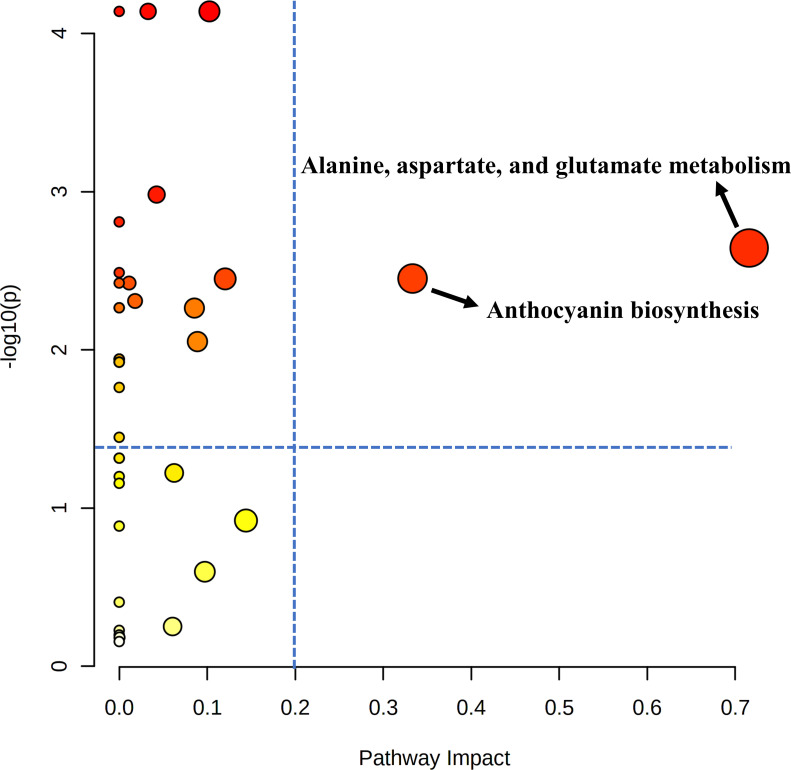
Pathway analysis of 2022 spring ‘Earlyyou’ grown on open-field or with shading treatment. Equivalent of cyaniding-3-glucoside was used as a respresentative for anthocyanin for pathway analysis. The blue dotted line indicates the standard at –log10(p) = 1.3, pathway impact = 0.2. .

Pathway analysis was conducted to find out which metabolic pathways were significantly changed by additional shading treatment under the APV system in ‘Earlyyou’ broccoli floret in spring 2022 (**Figure 5**). As a result, it was found that there were differences in alanine, aspartic acid, and glutamic acid metabolism and anthocyanin biosynthesis pathways (–log_10_(p) > 1.3, pathway impact > 0.2). The total amino acid content was significantly higher in shading treatment groups except for ‘Youil2ho’ cultivar of broccoli (**Figure S1**).

#### 3.4.2 Glucosinolate content

**Figure 6:**
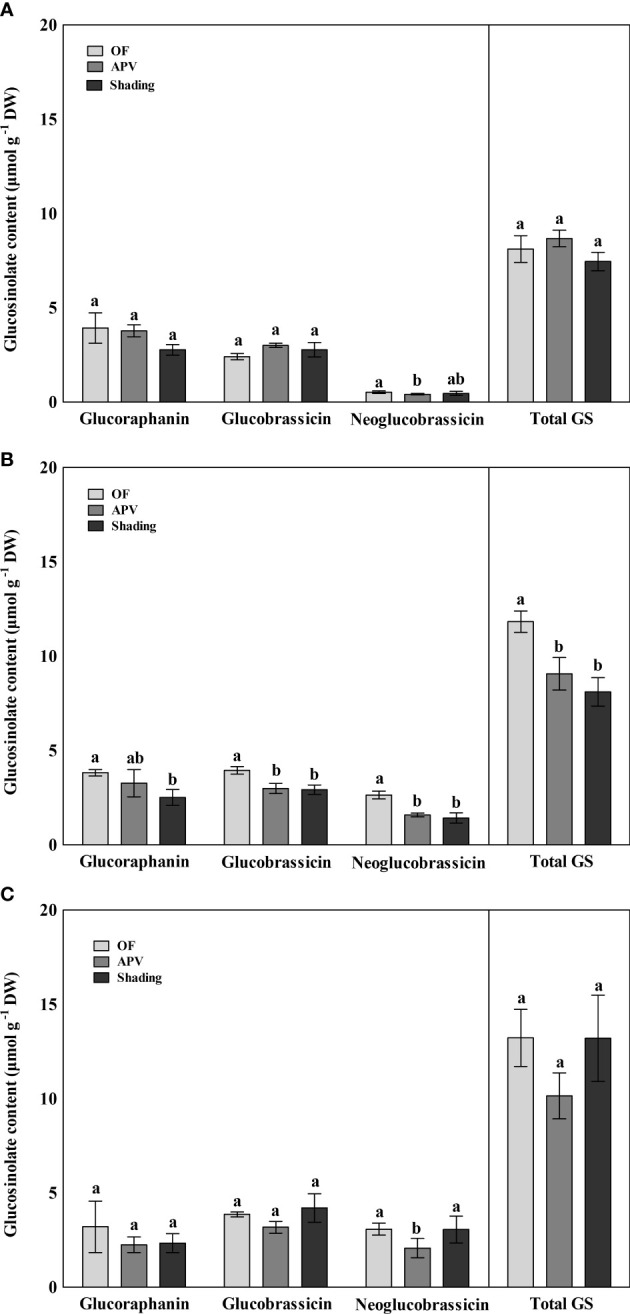
Glucosinolate contents of ‘Earlyyou’ of fall **(A)**, ‘Earlyyou’ of spring **(B)**, and ‘Youil2ho’ of spring **(C)**. Lowercase letters indicate significant differences among treatments within the same cultivar and year by HSD Tukey’s test (*p* < 0.05).

The glucosinolate contents of broccoli were quantified, and the results indicated three major or significantly changed individual glucosinolates and total glucosinolate (**Figure 6**). In the fall of 2021, neoglucobrassicin levels of ‘Earlyyou’ grown using the APV system were significantly lower than those grown on OF, whereas the two other glucosinolates or total glucosinolate levels did not significantly differ among the treatment groups. In the spring of 2022, the glucoraphanin content of ‘Earlyyou’ was significantly lower in plants grown with shading treatment than that in plants grown on OF. Glucobrassicin and neoglucobrassicin levels in ‘Earlyyou’ grown using the APV system and with shading were significantly lower compared with the OF group. Total glucosinolate levels were decreased by the APV system and shading treatment. In the case of ‘Youil2ho’, almost none of the glucosinolate compounds showed a significant difference among the groups, except neoglucobrassicin in the APV group.

#### 3.4.3 Chlorophyll and total anthocyanin content

**Figure 7:**
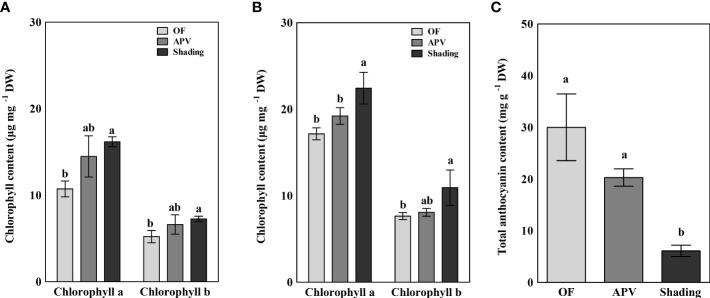
Chlorophyll a and b contents extracted from the buds of 2022 spring ‘Earlyyou’ **(A)** and ‘Youil2ho’ **(B)**. Anthocyanin abundance was measured only in the ‘Earlyyou’ cultivar **(C)**. The content of total anthocyanin was calculated as cyanidin-3-glucoside equivalent. Lowercase letters indicate significant differences among treatments within the same cultivar and year by HSD Tukey’s test (p < 0.05).

The chlorophyll *a* and *b* contents of ‘Earlyyou’ and ‘Youil2ho’ grown in the spring of 2022 were calculated (**Figures 7A, B**). Shading treatment of the ‘Earlyyou’ cultivar resulted in significant increases in both chlorophyll *a* and *b* levels compared with the OF group, whereas there were no significant differences between the APV group and the other two groups. Similar results were obtained for the ‘Youil2ho’ cultivar, except for the difference between the APV and shading treatment groups in chlorophyll *a* content. The results of the total anthocyanin content assessment equilibrated by cyanidin-3-glucoside levels are shown in **Figure 7C**. The total anthocyanin content was significantly lower in the shading treatment group (6.1 mg per grams of dry weight) compared with the OF (30.0 mg^-1^g DW) and APV (20.3 mg^-1^g DW) groups.

## 4 Discussion

The decrease in solar radiation is the common change of microclimate in APV systems. Since an APV system installed on OF does not constitute a closed system, it would not be expected to affect the atmospheric conditions (Marrou et al., 2013; Touil et al., 2021). However, this does not necessarily mean that the growth conditions do not change in an open environment. It was reported that due to low vapor pressure deficit and high soil moisture induced by APV systems, water could be saved in the dryland (Barron-Gafford et al., 2019). The water content was measured by soil sensors located 20 cm below the soil surface in this study, whereas the previous works in the literature measured it at 5 cm below the surface. This suggests that the difference in water content between OF and APV systems was underrated, because the irradiation decreases as one moves deeper under the ground. Despite the equal sensor depth in soil, in the spring of 2022, water content in the APV system was higher than that in OF due to several reasons. Within the period of shading, it was cloudy and rainy because of the plum rain called East Asian rainy season. Distribution of rainwater reaching the ground was not even, and it was higher under panels (Weselek et al., 2021). Thus, it is possible that the soil sensor was placed in an area where rainwater fall along the PV panel. Even when the air temperature did not change, the soil temperature varies due to the APV structure or additional shading treatment. According to the literature, soil temperature levels in APV systems are about 1.9°C and 2.2°C lower than those in OF when measured 5 and 25 cm below the surface, respectively (Marrou et al., 2013). Another study reported that the difference in the soil temperature between OF and APV systems is 1.2°C (Weselek et al., 2021). Soil temperature was about 1°C lower in the case of shading treatment due to reduced infrared ray reaching to the surface.

Too much shading might evoke significant decreases in the yields of broccoli. Our previous work suggested that the average weight of broccoli grown using an APV system is decreased; however, it does not differ significantly compared with plants grown on OF (Chae et al., 2022). The yields of several types of crops according to the degree of shading or light reduction have already been reviewed in previous reports (Weselek et al., 2019; Touil et al., 2021; Zainol Abidin et al., 2021). Such reports indicated that the yield does not significantly decrease when panels cover about 20%–30% of the total cultivation area. However, with more than 50% shading, the yield decreases significantly. In any case, the average head weight decreased regardless of cultivar or season in our study. In the spring of 2022, the mean weight decreased by more than 20% in all treatments except in the case of shading treatment of ‘Earlyyou’. The criteria for how much yield reduction will pose threat to food security is ambiguous. However, the 20% figure is certainly not to be overlooked. The following solutions may be suggested for concerns about decreases in yield. Delaying harvest or early transplanting of seedlings may help minimize the decreases in yield. Next, APV systems have basic structures and electrical facilities; thus, they can also be equipped with a system that automatically provides shading when the light intensity limit is exceeded. Finally, scattering incoming light through diffusors is another method to reduce the loss in PPFD while preventing exposure to strong direct light.

Despite the decreases in yield, crops grown using the APV system may have a positive apparent quality compared with the those grown on the OF. Usually, consumers prefer broccoli with more greenish head. Coles (2016) found that consumers also prefer dark-green broccoli compared with purple broccoli. According to the International Commission on Illumination (CIE), higher a* values in the color space indicate higher red content and the lower a* values indicate higher green content (Schanda, 2007). Therefore, as seen in **Table 2**, it is meaningful that ‘Earlyyou’ broccoli have a greenish color when grown using the APV system or with shading treatment compared with plants grown on OF. Likewise, low a* values (indicator of redness) are supported by photos shown in **Figure 7**. The hue angle, calculated using a* and b*, is used as an indicator to measure the change in visual quality after harvesting (Schonhof et al., 2004; Aiamla-Or et al., 2009; Ku et al., 2013). The hue angle of broccoli is about 130° just after the harvest and gradually drops with time, falling to 90° after 6 days (Aiamla-Or et al., 2009). In this study, the hue angle of broccoli was about 100–110°. ‘Earlyyou’ cultivated with shading treatment had a low value of a* and a high hue angle when the head started to extend. It is noteworthy that a high hue value was obtained when ‘Youil2ho’, a cultivar that does not express anthocyanin, was grown using the APV system or with shading. This suggests that even the broccoli of the green cultivar may have a greener color through shading.

In this study, we collected the data of all the metabolites (water-soluble metabolites, glucosinolates, and amino acids) analyzed and conducted a PCA and pathway analysis to identify the pathways that were altered by the APV system or shading treatment. Both the autumn and spring seasons of the ‘Earlyyou’ cultivar showed elevated levels of several amino acids upon shading treatment. In particular, glutamine, asparagine, aspartic acid, and 5-oxoproline levels were high in plants receiving the shading treatment regardless of the season. Aspartic acid was the only compound that significantly changed upon growth using the APV system or with additional shading treatment in both *B. oleracea* plants. 5-Oxoproline was significantly accumulated in additional shading-treated broccoli in both spring and fall seasons. A previous study on green tea also reported that shading treatment affects 5-oxoproline content (Ku et al., 2010). Independent of the crop type, aspartic acid content in plants grown with shading treatment was relatively higher than those in plants grown on OF. It has been known that shading to green tea results in good visual quality of leaf and high amino acid content related to umami like theanine but low content of caffeine and catechin responsible for bitter taste (Ku et al., 2010; Sano et al., 2018; Unno et al., 2020). High content of several amino acids in plants grown with shading treatment suggests that some metabolic processes have altered. Thus, it may be a good idea to test for potential changes in the taste of broccoli due to shading treatment in future studies.

Glucosinolate is one of the secondary metabolites that contain sulfur and is mainly found in *Brassicaceae*. In broccoli, glucoraphanin is the highest in aliphatic glucosinolate, and there are also indole glucosinolates such as glucobrassicin and neoglucobrassicin (Radošević et al., 2017; Ilahy et al., 2020). In the spring of 2022, total glucosinolate contents of ‘Earlyyou’ grown using the APV system and with shading were lower than those of the plants grown on OF; however, in the fall of 2021, no significant differences were observed among the treatment groups. Similar differences were found in individual glucosinolates such as glucoraphanin and glucobrassicin. Glucoraphanin has been known as one of the important glucosinolates in broccoli because its hydrolysis product, sulforaphane, has high anticancer activity (Kamal et al., 2020). Therefore, the decrease in glucoraphanin content in spring of 2022 by additional shading treatment (2.52 ± 0.42 μmol g^-1^ DW) compared with the OF group (3.80 ± 0.17 μmol g^-1^ DW) was observed, whereas no significant glucoraphanin loss was detected in broccoli grown under the APV system. On the other hand, APV has a significant loss of sulforaphane by analysis of glucosinolate hydrolysis product in **Figure S2**. Erucin, the hydrolyzed product of glucoerucin showed similar tendency to sulforaphane. 3-Butenyl isothiocyanate, a hydrolysis product of gluconapin, was significantly reduced in the shading treatment, but there was no difference in APV. Thus, it is required that optimal vegetable production practice for additional shading treatment should be further investigated to minimize health promoting compounds and yield. Potentially, it is required to transplant early seedlings during spring production to avoid hot weather. It is possible to harvest little late or delayed harvest practice to minimize yield loss. The biggest difference between plants grown on OF and those grown using the APV system or with shading is the light intensity; thus, the change in glucosinolate was probably due to this difference. High irradiation, especially UV radiation, induces high content of total glucosinolate or individual glucosinolates. Individual and total glucosinolates are increased by UV-A and UV-B treatments in broccoli sprout (Moreira-Rodríguez et al., 2017). In addition, broccoli grown on OF was exposed to higher temperatures compared with those grown using the APV system or with shading treatment due to infrared radiation. It was reported that total glucosinolate levels in plants under high day and night temperatures (21°C and 15°C, respectively) are higher than those in plants under relatively low temperatures (15°C and 9°C, respectively) (Steindal et al., 2013). In addition, along with the UV effect described above, the increase in glucosinolate levels by environmental stress has been reported in several literature reviews (Del Carmen Martínez-Ballesta et al., 2013; Moreira-Rodríguez et al., 2017). However, in this study, ‘Earlyyou’ was cultivated in both fall and spring, and there was a significant difference only in spring. Unlike the fall harvest season, which gets colder as the harvest season approaches, the high temperature levels of the spring harvest season might have induced the high levels of glucosinolates.

Since chlorophyll or anthocyanin is concentrated in the bud part of the broccoli head, only the buds were separated to measure these pigment contents. Chlorophyll is attributed to mainly green color, whereas anthocyanin is attributed to red or purple color. The former is found in almost every part of the plant and is involved in photosynthesis, and the latter serves to defend against damages from various stresses (Humphrey, 2004; Wrolstad, 2004). UV-B is one of the stress factors and affects the expression of various gene groups (Ballaré, 2003). Chlorophyll content was increased by shading treatment regardless of chlorophyll type and broccoli cultivar. On the other hand, the anthocyanin content of ‘Earlyyou’ significantly decreased in response to shading treatment (**Figure 6**). Lee et al. (2013) showed that chlorophyll *a* levels are nearly tripled compared with controls to absorb more light when the tea trees are 95% shaded for 15 days before harvesting. Zhu et al. (2017) reported that chlorophyll *b* abundance is increased to improve photosynthesis, whereas anthocyanin biosynthesis is inhibited by low light conditions on purple pak choi. Light is a major stimulus factor for inducing anthocyanin synthesis in plants (Liu et al., 2018b). Liu et al. (2020) reported that in the case of purple broccoli, anthocyanin content is lower when only the head is shaded than that when only the leaves are shaded. They also revealed that broccoli head is the main photo-response receptor for anthocyanin synthesis and identified the genes involved in the regulation of anthocyanin synthesis (*BoMYB6-1*, *BoMYB6-2*, *BoMYB6-3*, *BoMYB6-4*, *BoTT85-1*, *BoTT85-2*, and *BoEGL5-3*) as well as the structural genes (*PAL1*, *4CL-1*). The high chlorophyll content in the shading environment may be due to increased efficiency of photosynthesis and is consistent with the high hue value of broccoli head. It can be postulated that shading treatment reduces the UV exposure, resulting in reduced stress and low expression levels of anthocyanin-related genes. In addition, due to additional shading, the anthocyanin content was one-fifth of that observed in plants grown on OF, which is consistent with the decrease in a* value and the greener appearance. In the principal component analysis indicated that aspartic acid content was significantly affected by shading treatment, regardless of season or cultivar. Aspartic acid is involved in various biosynthesis processes as a precursor for biosynthesis (De La Torre et al., 2014). The derivatives of aspartic acid include phenylalanine and glutamic acid, which are the precursor metabolites of anthocyanin and chlorophyll biosynthesis, respectively. Thus, it is possible that the lower levels of anthocyanin accumulation due to shading may result in a surplus of its precursor. Alternatively, it is possible that increased chlorophyll biosynthesis induced its precursor to meet the demand, which required further study. Conclusively, it is possible that accumulation of aspartic acid serves as a biomarker of shading treatment under APV grown vegetables. Chlorophyll accumulation and anthocyanin reduction by shading treatment was previously reported; however, this study is the first report demonstrating a significant alteration in alanine, aspartate, and glutamate metabolism as a result of cultivation using an APV system or additional shading treatment in APV systems.

## 5 Conclusion

We evaluated several metabolites and color properties of broccoli grown with additional shading under the APV system. Concentrations of several amino acids and glucosinolates were affected; especially the amino acids exhibited elevated levels upon additional shading treatment. Even under the same shading conditions, glucosinolate contents varied according to the season and among the cultivars. Additional shading significantly improved color properties as demonstrated by changes in redness and hue angle. The anthocyanin content of broccoli grown under additional shading was reduced to one-fifth of that in broccoli grown on OF. Furthermore, the chlorophyll content, responsible for the greener color, was increased by additional shading regardless of the cultivar. Our findings suggest that APV systems can be utilized to improve crop quality.

## Data availability statement

The original contributions presented in the study are included in the article/**Supplementary Material**. Further inquiries can be directed to the corresponding author.

## Author contributions

H-WM: conceptualization, methodology, validation, formal analysis, investigation, writing—original draft preparation. K-MK: writing—review and editing, methodology, supervision, project administration. All authors contributed to the article and approved the submitted version.
