# Supplementary material for: The effect of additional shading utilizing agriphotovoltaic structures on the visual qualities and metabolites of broccoli
Source: Front Plant Sci. 2023 Mar 3;14:1111069. doi: 10.3389/fpls.2023.1111069 (PMC10020647; doi:10.3389/fpls.2023.1111069)
Supplement: Supplementary file 1 [file DataSheet_1.docx]

**The effect of additional shading utilizing agriphotovoltaic structures on the visual qualities and metabolites of broccoli**

Hyeon-Woo Moon ^1^ and Kang-Mo Ku ^1,2*^

^1^ Department of Horticulture, Chonnam National University, Gwangju 61186, Republic of Korea;

notorious931216@gmail.com

^2^ Department of Plant Biotechnology, Korea University, Seoul 02841, Republic of Korea

* Correspondence: ku_km@korea.ac.kr

**Appendix**


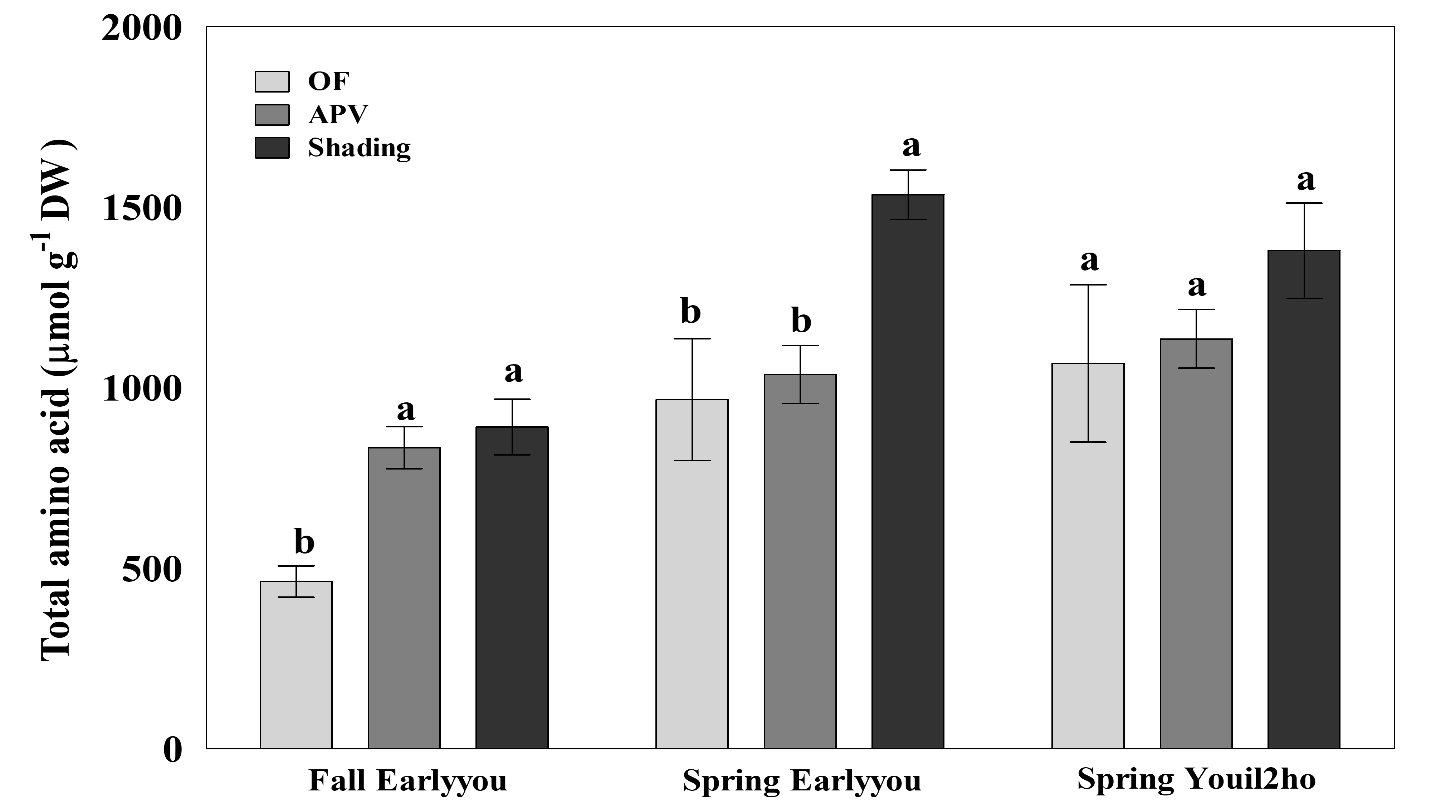


**Fig S1.** Total amino acid content of broccoli “Earlyyou” in the spring of 2022. Lowercase letters indicate significant differences among treatments by HSD Tukey's test (*p* < 0.05).


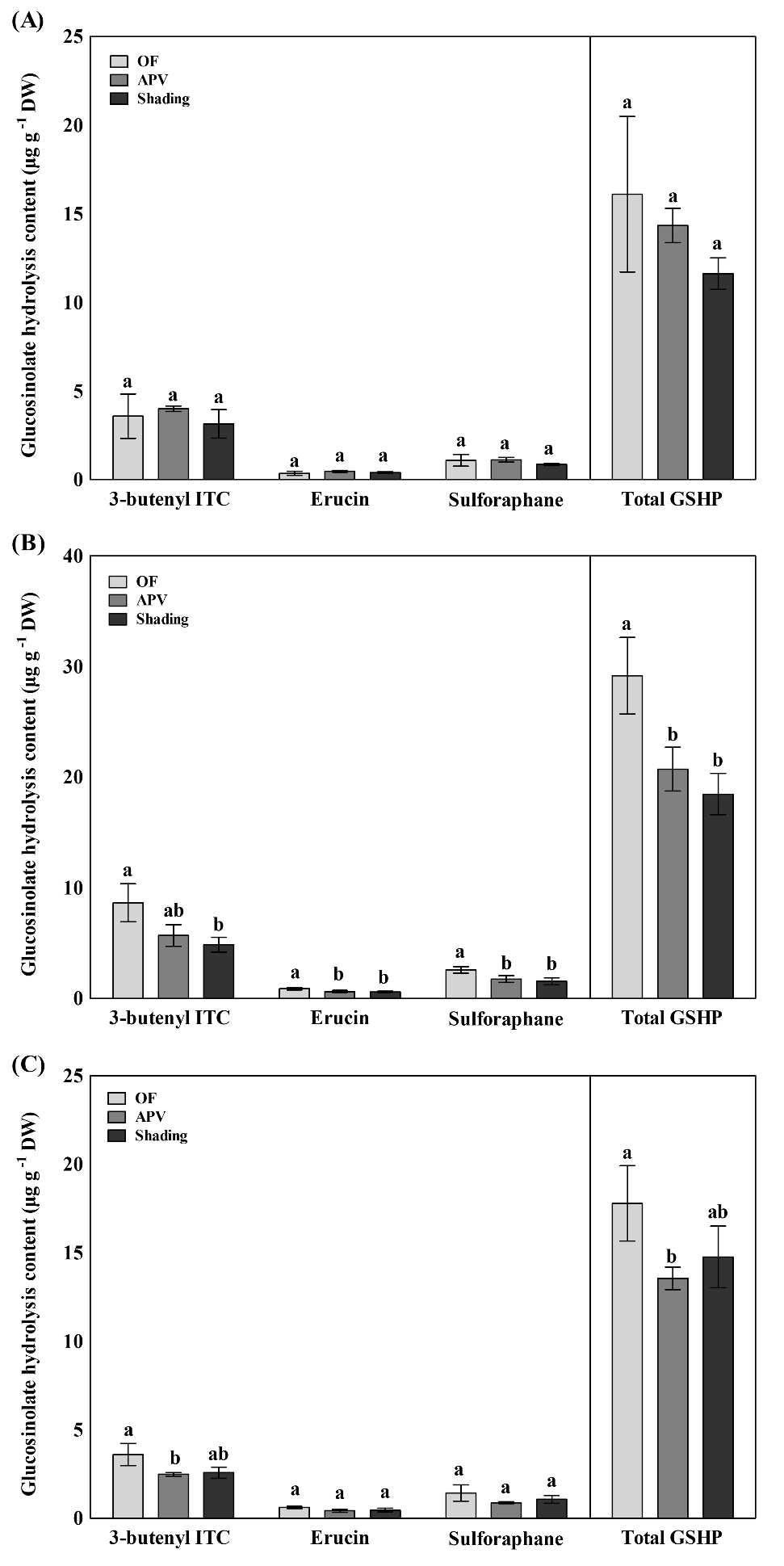


**Fig S2.** The content of glucosinolate hydrolysis products (GSHP) of “Earlyyou” of fall (A), “Earlyyou” of spring (B), and “Youil2ho” of spring (C). Three isothiocyanate products (3-butentyl isothiocyanate, 3-butenyl ITC; 4-methylthiobutyl isothiocyanate, Erucin; 4-(methylsulfinyl)butyl isothiocyanate, Sulforaphane) were identified as major GSHP of broccolis. Products were quantified as phenyl isothiocyanate equivalent. Lowercase letters indicate significant differences among treatments by HSD Tukey's test (*p* < 0.05).
